# The Evaluation of Various Restoration Techniques on Internal Adaptation of Composites in Class V Cavities

**DOI:** 10.1155/2014/148057

**Published:** 2014-10-02

**Authors:** D. Dionysopoulos, C. Papadopoulos, E. Koliniotou-Koumpia

**Affiliations:** Department of Operative Dentistry, School of Dentistry, Aristotle University of Thessaloniki, 54124 Thessaloniki, Greece

## Abstract

*Aim.* The aim of this study was to evaluate the effect of different restoration techniques on the formation of internal microgaps between materials and dentin in class V restorations. *Materials and Methods.* Twenty-five extracted human premolars were prepared with standardized class V cavity outlines (3 mm × 2 mm × 2 mm). The cavities were randomly divided into 5 groups of 10 cavities each and restored according to manufacturer's instructions: Group 1: preheating (55°C) conventional composite (Filtek Z250), Group 2: flowable composite (Filtek Flow), Group 3: Filtek Flow + Filtek Z250 light-cured separately, Group 4: Filtek Flow + Filtek Z250 light-cured simultaneously, and Group 5 (control): Filtek Z250 at room temperature (23°C). The specimens were then thermocycled and cross-sectioned through the center of the restoration. Subsequently, impressions were taken, and epoxy resin replicas were made. The internal adaptation of the materials to the axial wall was analyzed under SEM. *Results.* The preheated Filtek Z250 (Group 1) showed better internal adaptation than the room temperature groups (*P* < 0.05). The combination of Filtek Flow with Filtek Z250 which was light-cured separately (Group 3) exhibited better internal adaptation than control group (*P* < 0.05). *Conclusion.* Different restoration techniques exhibit different behavior regarding internal adaptation to dentin after photopolymerization.

## 1. Introduction

Dental composite resins are the most frequently used direct tooth-colored restorative materials restoring cervical lesions. Adaptation of the restorative materials to cavity margins and internal cavity surfaces are crucial for long-term performance of restorations, especially for cavities with high configuration factor such as class V cavities [1]. The shrinkage of composite resins during photopolymerization induces stresses at the tooth/restorative interface and as a consequence may cause failures in the bond, generating gap formation (10–15 *μ*m) [2, 3]. Such microgaps are considered deleterious because they allow the transit of fluid or bacteria between the dentin pulp complex and the oral environment, leading to postoperative sensitivity and secondary caries formation [4].

The magnitude of interfacial stress depends on substrate variables like cavity configuration (C-factor) or compliance of the tooth. On the other hand, stress at the adhesive interface can be controlled by material properties and restoration technique [5]. Approaches to minimize the adverse effects of photopolymerization shrinkage and gap formation have primarily focused on incremental placement of the material [6], use of soft-start light-curing units [7], use of semidirect and indirect restorations [8], and placement of liners with a low modulus of elasticity as stress relievers such as flowable composite resins [9].

A number of studies, showing improved margin adaptation with flowable liners, have attributed to their results to the reduced viscosity of the materials, allowing them to wet better the walls of the prepared cavity [10–12]. However, the reduced viscosity of flowable composite resins is achieved by lowering the filler content, and as a result these materials exhibit higher polymerization shrinkage and lower strength than conventional composite resins [13].

It has been suggested that by preheating conventional composite resins around 55–60°C, a transient viscosity reduction comparable to that of flowable composite resins can be obtained. Thus, clinicians could benefit from using only a single material during the restorative procedure, which produces lower polymerization shrinkage and provides greater wear resistance [14]. However, the benefit of using preheating composites instead of using flowable liners is still debatable.

The aim of this* in vitro* study was to evaluate the effect of different restoration techniques on the formation of internal microgaps between materials and dentin of the axial wall in class V restorations after photopolymerization. The null hypothesis of the study was that there is no significant difference among restorative techniques in microgap width formation at the interface between the materials tested and the dentin substrate.

## 2. Materials and Methods

The materials used in this study were a microhybrid composite resin (Filtek Z250), a flowable composite resin (Filtek Flow), and an “etch and rinse” two-step adhesive system (Adper Scotchbond 1 XT). Their technical characteristics are shown in Table 1. Twenty-five freshly extracted human premolars for orthodontic reasons were selected, cleaned, and stored in a solution of 0.5% chloramines at 4°C until used. To ensure that the teeth were free of cracks, defects, or caries, they were examined under ×10 magnification by means of optical microscope.

Two standardized class V cavity preparations for each tooth (total 50 cavities) were made with a no. 245 carbide bur on the buccal and lingual surfaces using a high-speed handpiece with water coolant. The preparations included an occlusal margin in enamel and a gingival margin in dentin. The dimensions of the cavities were 3 mm wide, 2 mm high, and 2 mm deep. The burs were replaced with new ones after every fifth preparation. The preparation dimensions were measured with a digital caliper for width and a periodontal probe for depth. The teeth were randomly assigned into 5 groups (10 cavities each) and restored with a combination of the tested materials as indicated in Table 2.

After etching with 35% phosphoric acid of enamel for 30 sec and dentin for 15 sec, the cavities were thoroughly rinsed with water for 15 sec and the adhesive Adper Scotchbond 1 XT was applied to the cavity walls. A gentle air-drying of the cavities was followed to remove excess solvent and the adhesive light-cured for 10 sec with a QTH light-curing unit (Elipar 2500, 3M ESPE, USA) at 1400 mW/cm^2^. The restorations in all groups were made according to manufacturer's instructions and the preparations of the cavities and restorations were made by one operator. A commercially available unit (ENA Heat, Micerium SpA, GE, Italy) was used to preheat the composite resin prior to its application into the cavities in Group 1. The control group of the study was Group 5 (Filtek Z250, 23°C).

The restorations were finished after 24 h with finishing diamond burs and polishing discs (Sof-Lex, 3M ESPE, USA) of decreasing abrasiveness. The specimens were stored at 37°C for 7 days in saline solution and then the teeth were subjected to 800 cycles between temperature baths at 5°C and 55°C with a dwell time of 30 sec. Each specimen was sectioned in half through the center of the restoration with a slow speed saw (Isomet 1000, Buhler Ltd., Lake Bluff, IL, USA) at 300 rpm, resulting in two fragments. The fragments polished down using decreasing grit abrasive silicon carbide papers (600 and 1200 grits).

Subsequently, each half was sectioned along the longitudinal axis through the center of the restorations to obtain a slice of 2 mm in thickness. In order to remove the grinding debris, the specimens ultrasonicated in saline solution for 20 sec. After being slightly air-dried, impressions were performed with vinyl polysiloxane material, which served as molds to fabricate epoxy resin replicas (Epofix resin, Struers Tech A/S, Denmark), reproducing the interface between dental tissues and tested materials.

The specimens were mounted on aluminum stubs, sputter-coated with carbon to a thickness of approximately 200 Å in a vacuum evaporator (at low vacuum), and examined under scanning electron microscope (JEOL Ltd., JSM-840, Tokyo, Japan) at 19 KV. Photomicrographs were performed with ×500 magnification in the area of the largest microgap width. The width of the internal gap on axial wall was measured and the mean gap width for each group was computed. Ten measurements of microgap width were carried out for each experimental group by two independent researchers who were unaware of the group of the tested specimens and each other's measurements. Statistical analysis of the data was made using one-way ANOVA, Duncan's test, and Kruskal-Wallis test at a level of significance of *a* = 0.05.

## 3. Results

The mean width and standard deviation of internal gaps (*μ*m) obtained from each experimental group between dentin and the materials tested are shown in Table 3. The microgaps were consistently observed particularly in all experimental groups. The percentages of gap free interfaces of the specimens observed in each experimental group are presented in Table 3. The results indicated that the specimens restored with preheated composite resin (Group 1) exhibited lower mean width of gap formation than those restored with room-temperature composite resin in control group (Group 5), (*P* < 0.05). Moreover, the specimens of Group 3 (light-curing of flowable and conventional composite separately) presented lower mean width of internal gap than those of Group 4 (cocured), (*P* < 0.05). Specimens restored with flowable composite resin presented no statistically significant differences with specimens restored with room-temperature composite resin (*P* > 0.05).

**Figure 1:**
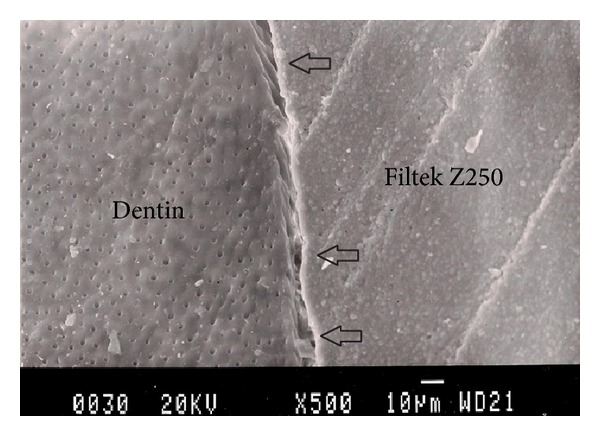
Representative SEM photomicrograph of a Group 1 specimen (Filtek Z250, 55°C). The arrows indicate microgap formation between Filtek Z250 and dentin.

**Figure 2:**
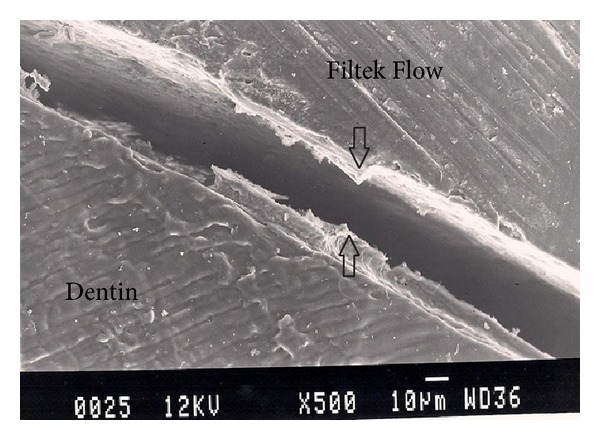
Representative SEM photomicrograph of a Group 2 specimen (Filtek Flow, in bulk). The arrows indicate large microgap formation between Filtek Flow and dentin.

**Figure 3:**
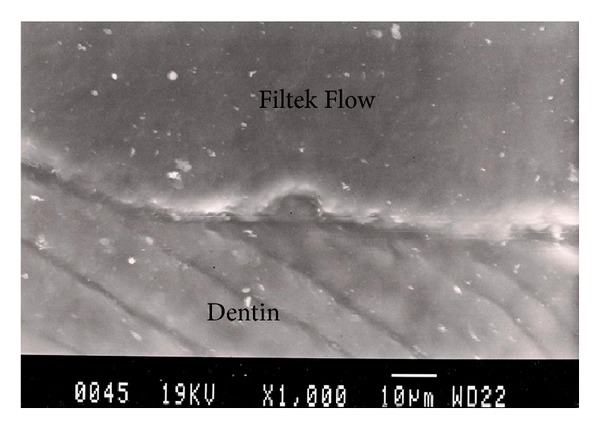
Representative SEM photomicrograph of a Group 3 specimen (Filtek Flow + Filtek Z250, light-cured separately). Good adaptation between Filtek Flow and dentin is observed.

**Figure 4:**
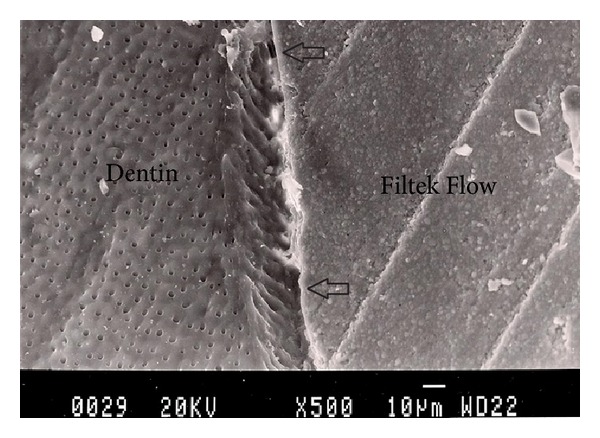
Representative SEM photomicrograph of a Group 4 specimen (Filtek Flow + Filtek Z250, light-cured together). The arrows indicate microgap formation between Filtek Flow and dentin.

**Figure 5:**
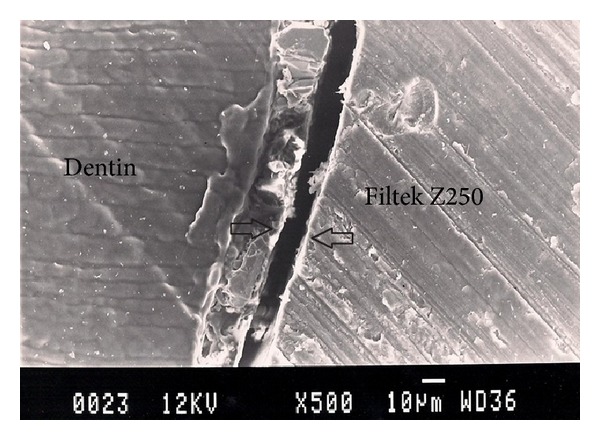
Representative SEM photomicrograph of a Group 5 specimen (Filtek Z250, 23°C). The arrows indicate microgap formation between Filtek Z250 and dentin.

Representative photomicrographs of each experimental group are presented in Figures 1, 2, 3, 4, and 5. The most common finding was the presence of microgaps in most specimens. The qualitative evaluation of internal adaptation revealed that continuous interfaces were achieved in several areas.

## 4. Discussion

The results of the present study demonstrate that there are statistically significant differences in the mean width of microgaps among the experimental groups. As a result, the null hypothesis of the study that there is no significant difference among restorative techniques in microgap width formation at the interface between the materials tested and the dentin substrate is rejected.

Previous studies have reported that placement of a flowable liner reduces the microleakage and increases the bond strength and fracture resistance values [15, 16]. Koliniotou-Koumpia et al. [17] reported that the use of a flowable composite as a liner 0.5 mm in thickness reduced microleakage and internal void formations and enhanced the internal adaptation as shown in the SEM in class V composite restorations. On the other side, various studies have shown that the placement of flowable composite liners does not have any beneficial effect on internal adaptation of restorations, due to low filler content and high polymerization shrinkage of resin liners [13, 18]. Consequently, the benefit of using flowable composites as liners to reduce microleakage and improve internal adaptation of the restoratives in cavity walls is still controversial, with studies showing improvement [19], no effect [5], and even deterioration [20] of the internal adaptation and microleakage. In the current study, the use of Filtek Flow as a liner when light-cured separately exhibited better behavior regarding gap formation than control group (restoration only with Filtek Z250).

The thickness of the flowable composite liner and whether the liner is polymerized prior to or simultaneously with the overlaying composite resin have also been investigated, with the hypothesis that less polymerization shrinkage is expected to occur in thinner liners [21]. Usually, the thickness of flowable liners which is recommended to be applied before conventional composite resin is 0.5–1 mm. Pecie et al. [13] reported that a 1 mm thick lining with an extremely low elastic modulus (2-3 GPa) could redistribute shrinkage stress as well as the use of a flowable composite did not significantly improve marginal adaptation of the restoration. In the present study, the use of 0.5 mm thick flowable liner improved internal adaptation of the restoration in comparison with the use of conventional composite resin alone.

Some authors have suggested a technique for flowable liners that involves injecting a small amount of flowable composite onto the floor of the cavity and the conventional composite resin is then put in place immediately and cocured simultaneously. This technique maintains a minimal volume of flowable composite, to increase cavity adaptation and reduce the potential for internal gap formation [17]. In the present study, this technique was applied in specimens of Group 4, which exhibited higher mean widths of internal gap than those of Group 3 (light-curing of flowable and conventional composite separately).

Other studies found reduction in microleakage with this combination compared with conventional techniques, which may be related to the lower modulus of elasticity in flowable composites [22]. The use of low modulus flowable composite resin may also increase the flexibility of the bonded assembly and may relieve the stress induced by the polymerization shrinkage of composites [23]. Materials with high elastic modulus destroy the bond between the restorative material and the tooth structure and lead to poor internal adaptation [24]. However, Oliveira et al. [9] found that using a flowable composite as liner or base material under composite resin restoration increases the polymerization shrinkage stresses at the adhesive interface leading to a possible adhesive failure. Furthermore, Sabatini et al. [10] reported that gap formation at the gingival margin of class II preparations was not improved relative to the control group by any of the placement methods tested in the present study.

In the current study, the use of preheating composite resin (Group 1) in class V restoration presented better internal adaptation than the use of the same composite resin in room temperature (Group 5). The flow characteristics of a restorative material affect its ability to adapt to the walls of a cavity preparation. Thus, a composite resin with a higher flow might adapt more easily the walls of a cavity preparation than one with lower flow values [25]. Many studies have recommended preheating of conventional composites instead of the use of a flowable composite liner. Wagner et al. [26] found that preheating a conventional composite resin resulted in significantly less microleakage at the cervical margins compared to a flowable liner and control. In another study, it has been found that under nonisothermal conditions (similar to a clinical situation) composite preheating enhanced marginal adaptation in the axial wall of the cavities [27]. Additionally, Daronch et al. [28] reported that flow of commercial hybrid composites can greatly increase upon preheating, but the extent of flow varies among brands and composite classifications. Moreover, the flowability of preheated composite materials never reaches the low levels of flowable composites [29].

In spite of these advantages, it has been found that preheating of composite resins may cause detrimental effects on the restoration margin as it increases the polymerization shrinkage of the composite resin [30]. In addition, Sabatini et al. [10] found that preheated composite resins did not significantly reduce gap formation at the gingival margin of class II restorations. According to the authors, once composite is pre-heated, there is a time delay between dispensing it from a syringe or campule, placing it into a preparation, contouring it, and subsequently light polymerizing it. It has been estimated that when a composite material is removed from the heating device, the temperature reduces 50% after 2 min and 90% after 5 min [28].

## 5. Conclusions

Within the limitations of this* in vitro* study the following statements can be concluded.

1. Different restoration techniques exhibit different behavior regarding internal adaptation to dentin.
2. The preheated composite resin shows better internal adaptation than the room-temperature composite resin.
3. The use of flowable composite resin as a liner when light-cured separately with the conventional composite resin shows better internal adaptation than the restoration with nonpreheated conventional composite resin.
4. The use of flowable composite resin as a restorative material shows similar internal adaptation to nonpreheated composite resin.

## Figures and Tables

**Table 1:** The materials used in the present study.

Material	Manufacturer	Type	Filler content wt%, vol%	Monomer composition
Filtek Z250	3M ESPE, Saint Paul, MN, USA	Microhybrid composite resin	77.6%, 60% zirconia/silica	Bis-GMA; UDMA; Bis-EMA; TEGDMA

Filtek Flow	3M ESPE, Saint Paul, MN, USA	Flowable composite resin	68%, 47% zirconia/silica	Bis-GMA; Bis-EMA; TEGDMA

Adper Scotchbond 1XT	3M ESPE, Saint Paul, MN, USA	Etch and rinse adhesive system	10% silica	Bis-GMA; UDMA; HEMA

**Table 2:** The experimental groups of the study.

Group	Type of restoration
1	Filtek Z250 (40 sec light-curing, 55°C, inserted in bulk)

2	Filtek Flow (40 sec light-curing, inserted in bulk)

3	Filtek Flow (0.5 mm layer at the axial wall, 20 sec light-curing) + Filtek Z250 (40 sec light-curing, 23°C)

4	Filtek Flow (0.5 mm layer at the axial wall, no light-curing) + Filtek Z250 (23°C) and co-light-curing for 40 sec

5 (control)	Filtek Z250 (40 sec light-curing, 23°C, inserted in bulk)

**Table 3:** Mean and standard deviation of internal gap (*μ*m) of the experimental groups.

Group	Mean width of internal gap (*μ*m)	Percentage of specimens with gap free interfaces
1	10.3 ± 2.2^A^	40%
2	22.5 ± 5.1^B^	10%
3	12.4 ± 2.4^A^	30%
4	18.2 ± 4.5^B^	10%
5	20.4 ± 4.3^B^	20%

Same letter indicates no statistically significant difference (*P* < 0.05).
